# Healthcare service disruption in 14 Latin American and Caribbean countries during the COVID-19 pandemic: Analysis of household phone surveys, 2020-2021

**DOI:** 10.7189/jogh.13.06023

**Published:** 2023-07-21

**Authors:** Cristian A Herrera, Amanda C Kerr, Julia M Dayton, Jakub J Kakietek

**Affiliations:** 1 World Bank, Washington DC, District of Columbia, USA; 2 Department of Public Health, School of Medicine, Pontificia Universidad Católica de Chile, Santiago, Chile; 3 Amsterdam Public Health Research Institute, Amsterdam UMC, University of Amsterdam, Amsterdam, Netherlands; 4 Department of Economics, University of Maryland, College Park, Maryland, USA

## Abstract

**Background:**

The coronavirus 2019 (COVID-19 pandemic) and associated responses have significantly disrupted healthcare. We aimed to estimate the magnitude of and reasons for households reporting healthcare disruption in 14 Latin America and the Caribbean (LAC) region countries from mid-2020 to mid-2021, and its relationship with country contextual factors.

**Methods:**

We used COVID-19 high-frequency phone surveys (HFPS) conducted in 14 LAC countries in three rounds in 2020 and one in 2021. We classified the reasons reported for healthcare disruption into four groups: concerns about contracting COVID-19, healthcare supply constraints, financial reasons, and public health measures (PHMs). We used bivariate and multivariate regressions to examine correlates of reported healthcare disruption with the above groups and country context as control variables.

**Results:**

On average, 20% of households reported a disruption in May-June 2020 (45% to 10% at country level), dropping to 9% in June-July 2020 (31% to 3%) and July-August 2020 (26% to 3%), and declining to 3% in May-July 2021 (11% to 1%). The most common reason reported for disruption was healthcare supply constraints, followed by concerns about contracting COVID-19, PHM, and financial reasons. In multivariable regression analyses, we found that a higher incidence of new COVID-19 cases (regression coefficient (β) = 0.018, *P* < 0.01), stricter PHM (β = 0.002, *P* < 0.01), fewer hospital beds per population (β = -0.011, *P* < 0.01), and lower out-of-pocket health spending (β = -0.0008, *P* < 0.01) were associated with higher levels of disrupted care. A higher care disruption was associated with a lower gross domestic product (GDP) per person (β = -0.00001, *P* < 0.01) and lower population density (β = -0.056, *P* < 0.01).

**Conclusions:**

Healthcare services for households in LAC were substantially disrupted during the COVID-19 pandemic. Findings about supply and financial constraints can inform the recovery of postponed healthcare services, while public health and contextual factors findings can inform future health system resilience efforts in LAC and elsewhere.

The coronavirus (COVID-19) pandemic and associated responses by different actors in society have disrupted healthcare in a significant way. The World Health Organization (WHO), by surveying key informants from June to November 2021, found that “nearly all countries are still affected by the COVID-19 pandemic with 92% of 129 countries (…) reporting some kind of disruption to services”, which was similar to what was reported in the first quartile of 2021 and the third quartile of 2020 [1].

From an individual’s perspective, a disruption of healthcare services occurs when an individual does not obtain care despite perceiving a need for it, representing the gap between perceived need and actual utilisation of services [2]. Need can be understood as “the capacity to benefit from healthcare”, particularly “appropriate” healthcare which is necessarily cost-effective [3]. Healthcare disruptions can either be postponed and provided later (e.g. immunisation) or forgone, meaning that they have been lost and cannot be replaced subsequently (e.g. stroke acute care) [4].

Globally, disruptions during the COVID-19 pandemic have been studied for childhood vaccine coverage [5], elective surgeries [6], hospital services for patients with cardiac diseases [7], and for several routine services [8-10]. In the USA, hospitalisations for acute cardiovascular conditions [11], emergency consultations [12], and prescriptions [13] were found to have been disrupted. In Europe, studies have shown disruptions in breast cancer management [14], health checkups [15], and general practice services [16]. In Latin America and the Caribbean (LAC), disruptions in cancer services have been documented for adults [17] and children [18]. Studies from Brazil have found reductions in medical appointments or hospital admissions for cancer care [19], stroke [20], prenatal procedures, diabetes, medical consultations [21], and reductions in cancer and cardiovascular disease care with a larger impact on women in Chile [22]. Nevertheless, recent literature reviews showed research on LAC is still lacking. A systematic review reported delays and disruptions to cancer services globally, but only two of the 62 included studies were from LAC [23], while a scoping review found similar trends on hospital services for cardiac disease patients with only four out of 92 studies including LAC countries [7].

Most studies analysing disrupted care have been performed from the perspective of healthcare providers and public health authorities using simulation methodologies [5,6], surveys to key informants [1,14], or administrative data [11,12,16]. Few have used population surveys; those that have were mainly from high-income countries [15,24,25], and fewer still used direct information from households [10]. Household data are particularly useful in low- and middle-income countries (LMIC) as they reflect the users’ perspective and experience and cover the deficiency of registration systems and data records, while providing information about households’ social and economic situation [26].

There is a critical evidence gap in direct reports from the general population about their use of needed healthcare services during the COVID-19 pandemic, particularly from LAC countries. Furthermore, trends over time, reasons behind delaying or postponing care, effect of the severity of the pandemic and policy responses to it, and influence of key contextual country factors have not been studied, and can inform strategies to recover postponed healthcare services, while shaping policy for supporting future health system resilience.

We aimed to estimate the magnitude of and reasons for households reporting disruption of needed healthcare services in 14 LAC countries in mid-2020 and mid-2021, and its relationship with country contextual factors. We also sought to explore the following research questions in the context of the COVID-19 pandemic: What was the magnitude of households’ reported disruption of healthcare services? What were the main reasons reported for the disruption? What was the relationship between the disruption of healthcare services and: COVID-19 burden, stringency of public health measures (PHM), out-of-pocket (OOP) health spending level in countries, and health system capacity? What have been the effects of key contextual country factors on reported healthcare services disruption?

## METHODS

### Data and study population

This cross-sectional study used previously collected household survey data from the LAC COVID-19 high-frequency phone survey (HFPS) supported by the World Bank in 2020 and the United Nations Development Programe (UNDP) in 2021. The HFPS were conducted in 14 countries in 2020 and 24 countries in 2021. For this study, we included all 14 countries with an HFPS in both years, representing almost 60% of the total regional population: Argentina, Bolivia, Chile, Colombia, Costa Rica, Dominican Republic, Ecuador, El Salvador, Guatemala, Honduras, Mexico, Paraguay, Peru, and Saint Lucia [27]. The survey was conducted in Spanish, except in Saint Lucia, where it was conducted in English.

Survey samples were based on a dual frame of cellphone and landline numbers generated through a random digit dialing (RDD) process, representative of households with a landline and households for which at least one member has a cellphone [28]. The first round was conducted between 8 May 2020 and 14 June 2020, the second from 5 June 2020 until 16 July 2020, the third from 5 July 2020 until 25 August 2020, and the fourth between May and July 2021. Eligible respondents were adults ≥18 years of age. Only one respondent per household was interviewed, answering on behalf of all members. The same respondent was interviewed in the first three rounds in 2020. The fourth round in 2021 revisited the same households in 12 countries with the aim of continuing the panel data set of households. The average panel household response rate was about 30%. Thus, to maintain representativeness in each country, the collection effort was complemented with a new sample as needed. For the other two countries, new households were interviewed (**Table 1** and Table S1 in the **Online Supplementary Document**).

**Table 1:** Description of surveys characteristics by country

Country	Number of female respondents, n (%)	Number of rural households, n (%)	Age of survey respondents, mean (SD)	Number of household members, mean (SD)	Number of households surveyed in 2020 wave one	Number of households surveyed in 2020 wave two	Number of households surveyed in 2020 wave three	Number of households surveyed in 2021 wave one
Argentina	2102 (59.38%)	384 (10.85%)	48 (16)	3 (3)	1001	694	629	1216
Bolivia	1787 (47.93%)	859 (23.04%)	37 (13)	5 (2)	1075	670	711	1272
Chile	1955 (55.57%)	664 (18.87%)	44 (15)	4 (2)	1000	622	684	1212
Colombia	2144 (59.74%)	840 (23.40%)	42 (14)	4 (2)	1000	730	638	1221
Costa Rica	1549 (53.41%)	1425 (49.14%)	41 (14)	4 (3)	801	636	658	805
Dominican Republic	1801 (53.73%)	784 (23.39%)	40 (15)	4 (2)	807	673	667	1205
Ecuador	2302 (51.65%)	1063 (23.88%)	40 (14)	4 (2)	1227	1025	853	1352
El Salvador	1401 (49.14%)	293 (35.82%)	39 (13)	4 (2)	804	625	604	818
Guatemala	1623 (49.57%)	491 (40.68%)	36 (13)	5 (2)	806	625	636	1207
Honduras	1619 (55.85%)	401 (39.28%)	36 (13)	5 (2)	807	550	521	1021
Mexico	4162 (58.18%)	1357 (18.97%)	46 (16)	4 (2)	2109	1245	1175	2625
Paraguay	1448 (52.96%)	555 (20.30%)	38 (13)	5 (3)	715	486	457	1076
Peru	2052 (52.97%)	903 (23.31%)	38 (14)	5 (2)	1000	841	821	1212
St. Lucia	1536 (54.35%)	1289 (45.58%)	49 (16)	4 (2)	1093	900	-	835
All Countries	27 481 (54.21%)	11 308 (25.29%)	41 (15)	4 (2)	14 245	10 322	9054	17 077

LAC – Latin America and the Caribbean, SD – standard deviation

### Disrupted healthcare

The primary outcomes of interest were self-reported disruption of healthcare services when perceived as needed and the reasons for the disruption [29].

Based on previous related studies [14,24,30] and the answer categories available in the questionnaire, we classified the reasons reported for healthcare disruption into four groups: 1) concerns about the threat of contracting COVID-19 (e.g. fear, anxiety), 2) healthcare supply constraints, including having no medical staff or no appointments available, closed facilities, not having enough supplies/tests or medication/drug, only treating emergencies, only treating COVID-19 patients, waiting for a long time, etc., 3) financial reasons due to lack of money or no medical insurance (e.g. to pay for services), and 4) PHMs implemented by governments, in particular stay-at-home orders, movement restrictions, and transportation interruption. These four categories allow for a better understanding of the barriers to accessing care during the pandemic, which can help the design of clinical and policy interventions targeted at them.

For the epidemiologic situation in each country, we used the number of new COVID-19 cases per million, as reported by Johns Hopkins University [31], using the number reported on the 15th of the month prior to the survey. For PHMs, we used the Oxford COVID-19 Stringency Index [32], applying the stringency index reported on the 15th of the month prior to the survey. From the World Bank’s Open Data [33], we extracted the latest OOP health spending as percentage of current health expenditure per country, along with the latest population-adjusted numbers of doctors, nurses, and hospital beds per country as measures of health system capacity indicators.

### Additional variables

We selected other control variables based on previous studies showing their relevance in relation to healthcare service disruption during the COVID-19 pandemic: gross domestic product (GDP) per person [34], rural population (percentage of total population in 2020) [35,36], population density (people per square kilometer of land area in 2020) [37], and age dependency ratio (% of working-age population) [38]. We obtained these data from the World Bank’s Open Data [33].

### Statistical analysis

We conducted descriptive analyses to present the characteristics of the study population and the frequencies of disrupted healthcare and reasons for disrupted healthcare for each country, and a population-weighted average for the 14 LAC countries.

We explored the bivariable correlation of reported disrupted healthcare with the four main areas of interest, using Pearson correlations and presenting *P-*values at 0.1, 0.05, and 0.01 levels, considering *P* < 0.05 as statistically significant.

We performed multivariable regressions to estimate the association of reported disrupted healthcare when needed as the dependent variable with the four main areas of interest, along with the selected control variables. We performed both ordinary least squares (OLS) and logistic regressions, but present only the OLS regression due to similarities in the results (Table S4 in the **Online Supplementary Document**). Our main results include detailed country level variables rather than country controls, allowing for further understanding of factors associated with the disruption of care. All regressions used robust standard errors. We applied the variance inflation factor (VIF) test to assess multicollinearity and determine the final model to be used. We dropped the variables exceeding a correlation of 70%.

We conducted data management and statistical analyses using R (version 4.1.1) and Stata software (version 15.1.).

## RESULTS

### Description of households

In most of the countries, the household respondent was a woman (54.2%), except in Bolivia, El Salvador, and Guatemala. They were 41 years of age, on average. Most households were in urban areas (74.7%) and had four household members on average (**Table 1**).

### Reported healthcare disruption and reasons for it

**Figure 1:**
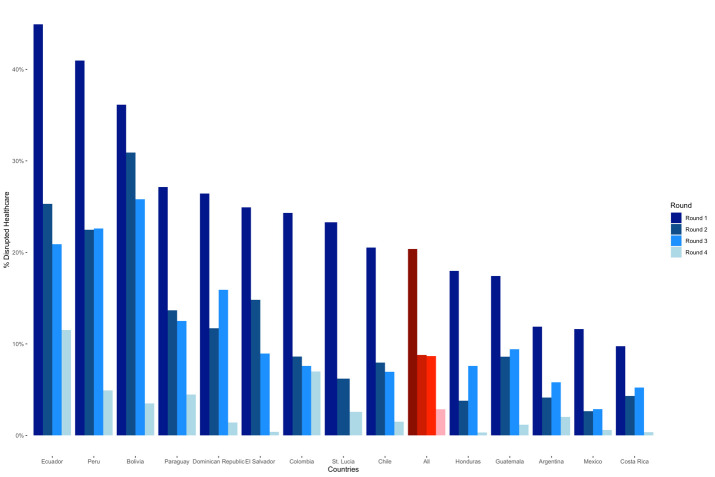
Share of households reporting needing healthcare whose healthcare services were disrupted in 14 Latin America and the Caribbean (LAC) countries, 2020 and 2021.

The largest disruption occurred in the first round of 2020; an average of 20.4% of households reported a healthcare disruption, ranging from 44.9% in Ecuador to 9.7% in Costa Rica. This dropped to an average of 8.8% and 8.7% in rounds two and three of 2020, respectively, and to 2.9% by round four in 2021. Ecuador, Peru, and Bolivia experienced the highest disruption across the four rounds, while Costa Rica, Mexico, and Argentina registered the lowest (**Figure 1**).

The most common reasons for care disruption were factors related to healthcare supply constraints, reaching above 50% of all reports in each round. Concerns about contracting COVID-19 followed, with 20.0% reporting this reason in the first round, declining to 5.6% in round four. Financial reasons oscillated between 3.7% and 12.4% of all reports. PHMs represented 15.5% in the first round and declined to 5.6% in the fourth round (**Table 2**). This distribution of reasons was similar in all countries (Table S2 in the **Online Supplementary Document**).

**Table 2:** Reasons for disrupting healthcare services, four survey rounds 2020 and 2021, average of 14 LAC countries (weighted by population)*

Round/Reasons	All reasons	Concerns about contracting COVID-19	Public health measures	Financial concerns	Healthcare supply constraints	Other reason
1	20.38%	4.07%	3.16%	0.75%	10.40%	2%
2	8.80%	1.94%	0.83%	0.84%	4.68%	0.50%
3	8.67%	1.77%	0.56%	1.07%	4.65%	0.61%
4	2.85%	0.16%	0.16%	0.35%	1.74%	0.46%

*Of the households that needed and did not receive healthcare, data on reasons for disrupted care were available for: 1084 households in round one, 351 in round two, 350 in round three, and 183 in round four.

### Correlation of reports of healthcare disruption with contextual factors

**Table 3** shows the bivariable correlation of households reports of disrupted healthcare with the four groups of reasons.

**Table 3:** Bivariable correlation of household reports of disrupted healthcare with incidence of new COVID-19 cases, Oxford COVID-19 Stringency Index, number of hospitals beds per 1000 population, and OOP health expenditure as percentage of current health expenditure in the 14 LAC countries

Factors	Correlation coefficient
Log new COVID-19 cases per million population	-0.37 (*P* = 0.008)
Oxford COVID-19 stringency index	0.54 (*P* < 0.001)
Hospital beds per 1000 population	-0.14 (*P* = 0.64)
OOP health expenditure as % of current health expenditure 2018	-0.16 (*P* = 0.59)

OOP – out-of-pocket, LAC – Latin America and the Caribbean

The number of new COVID-19 cases (incidence) was found to be inversely correlated with the reported disruption of care in the 14 countries (*P* = 0.008). However, this significant association is mitigated when focusing on the number of new COVID-19 cases and report of healthcare disruption specifically due to concerns of getting infected (*P* = 0.08), suggesting it might be mediated by other factors (Figure S1 and S2 in the **Online Supplementary Document**).

Level of strictness of PHMs, as measured by the Oxford COVID-19 Stringency Index, was positively correlated with reported disruption of care in 14 LAC countries (*P* < 0.001). This correlation was sustained when exploring the relation between PHM strictness and report of healthcare disruption due to PHMs in study countries (*P* < 0.001) (Figure S3 and S4 in the **Online Supplementary Document****).**

We did not find the correlation between health system capacity indicators and reported disrupted care to be statistically significant (*P* = 0.59). However, there was a significant association between OOP spending and healthcare disruption specifically due to financial concerns (*P* = 0.01) (Figure S5 and S6 in the **Online Supplementary Document**).

Similarly, we did not find the correlation between health system capacity indicators such as hospital beds per 1000 population and care disruption to be statistically significant (*P* = 0.64). This lack of correlation was maintained when examining the relationship between number of doctors, nurses, or hospital beds and disruptions specifically due to healthcare supply constraints (Figures S7-S12 in the **Online Supplementary Document**).

The OLS regression results, after applying the VIF multicollinearity test, show that healthcare disruption was higher when incidence of COVID-19 cases was higher, PHM were stricter, OOP health spending was lower, number of hospital beds was lower, GDP per person was lower, and population density was lower (**Table 4**). As an example of coefficients interpretation, a 100% increase in new COVID-19 cases generates a 1.8 percentage point increase in the probability of disrupting needed care, holding all else equal (see Table S3 and S4 in the **Online Supplementary Document** for correlation matrix and multivariable regression analyses).

**Table 4:** Ordinary least squares regression analysis of healthcare disruption in 14 LAC countries*

Factors	Healthcare not received	Robust standard errors	*P*-value
Log new COVID-19 cases per million population	0.01846†	0.00318	0.00000
Oxford COVID-19 stringency index	0.00155†	0.00023	0.00000
Hospital beds per 1000 population	-0.01126†	0.00276	0.00004
OOP health expenditure as % of current health expenditure in 2018	-0.00077†	0.00028	0.00547
GDP per person (current US$) in 2020	-0.00001†	0.00000	0.00000
Population density (people per square kilometer of land area) in 2020	-0.00012†	0.00004	0.00096
Constant	0.08045†	0.01726	0.00000
N	14 365		
*R* ^2^	0.097		

OOP – out-of-pocket, GDP – gross domestic product, LAC – Latin America and the Caribbean

*All specifications include year – month controls.

†Significance at 0.01 level.

## DISCUSSION

Using self-reported household-level data from nationally representative phone surveys in 14 LAC countries, we found that disruptions were the highest in the beginning of the pandemic (May-June 2020), with an average of 20.4% of households reporting a healthcare disruption when perceived as needed. Disruption declined to 8.8% and 8.7% in June-July 2020 and July-August 2020, respectively, and dropped further to 2.9% by May-July 2021. The most common reasons reported for care disruption were factors related to healthcare supply constraints (above 50% of all reports), followed by concerns about COVID-19 (between 5.6% and 20.0%), PHM (between 5.6% and 15.5%), and financial reasons (between 3.7% and 12.2%). The latter finding contrasts what has been found in low and low-middle income countries, where the main reason for disrupting care has been financial barriers [10].

Using bivariable and multivariable analysis, we found that the four main areas explored were correlated with substantial changes in healthcare disruption. Although the bivariable analysis showed an inverse relation, when controlling for other variables, a higher incidence of COVID-19 cases was associated with more disrupted care. This finding coincides with what other studies have shown in other regions [8,9]. A robust association between stricter PHMs and higher disruption of care across countries and time was found, consistent with what has been observed in European countries [34]. This can be explained by mobility restrictions and transport interruptions experienced in many countries. A higher health system capacity, particularly represented by hospital beds, was associated with less disruptions, meaning that countries with greater volume of healthcare managed to cope better. A higher country-level OOP health spending was associated with fewer disruptions, which might seem counterintuitive. However, during the COVID-19 pandemic, LAC health systems had both public and private sectors collaborating as never before. Since higher OOP is associated with larger private healthcare provision, this extended capacity and coordination with the public sector (for instance, through more hospital beds and telemedicine for ambulatory care) might have generated synergies that helped to reduce the extent of care disruptions.

Regarding contextual factors, countries with a relatively lower level of development (e.g. Ecuador, Bolivia, Paraguay) were most affected by disruptions, while countries with relatively higher level of development were less so (e.g. Costa Rica, Mexico, Argentina). This was further supported by the finding that higher GDP per capita was associated with lower levels of healthcare disruption in the multivariable analysis, suggesting an association between country income and health sector performance, possibly through both having a stronger health system and better population well-being. Additionally, countries with higher population density experienced lower levels of healthcare disruption, likely because higher density is usually an expression of urbanisation that normally facilitates healthcare access due to higher development of health systems and connectivity (e.g. public transport) in such geographical areas [37].

This analysis focused on disrupted healthcare at the country level to highlight contextual and policy drivers associated with disruptions across Latin American countries. Future analyses will explore differences within countries and the extent to which different household-level characteristics (e.g. household income, education level of respondent) affect the likelihood of healthcare disruptions.

### Limitations and implications for research

One important limitation of this study is the lack of pre-pandemic information about care disruption, impeding a better understanding and contextualisation of findings. Due to the nature of the survey questions, we cannot differentiate between postponed and forgone care nor type of services, which would better inform decision making. Regarding the data, although all survey rounds were nationally representative, the loss of panel households between 2020 and 2021 could create some noise in the information when comparing between these years. Despite including almost 60% of LAC population, the fact that HPFS was missing large countries such as Brazil (which was only available in the 2021 round) and Venezuela is a significant limitation. Another limitation is that one respondent reported the information for the whole household, which might result in less reliable reporting.

### Implications for policy and practice

In the short-term, as PHMs and concerns about COVID-19 are progressively declining in most LAC countries, healthcare supply constraints and financial barriers will be key to planning recovery of postponed healthcare services. For instance, use of digital tools for expanding capacity has been explored (e.g. online prescription renewals, telemedicine, digital mental healthcare) and service adaption to bring care closer to people with strategies such as home blood pressure monitoring, community delivery of drugs for patients with stable chronic conditions, weekend opening hours, and special vaccination campaigns [39]. Working with the private sector to expand provision while defining the appropriate regulation to achieve pre-defined public health goals and the best use of public resources will also be important [40]. However, recovery should also be responsive to differences in needs between persons with different diseases/conditions and health literacy. Monitoring fairness in access to and use of healthcare services during the period of recovery should be of concern for policy makers [41].

In the medium and long term, health system resilience in LAC countries could be boosted with investments that can strengthen health systems capacity (e.g. primary care, hospital beds, healthcare workforce, telemedicine home care), while developing structural transformations that can reduce health systems fragmentation and further coordinate public and private healthcare provision to achieve public health goals [41]. Renewing public health emergency preparedness and response plans can help to have a better application of PHM in future epidemics/pandemics aiming to control outbreaks while minimising its impact of routine care provision. Finally, development efforts in LAC countries should continue, including for the preparation of social and health systems to protect vulnerable population (e.g. elderly) and address the urban/rural divide [42,43].

## CONCLUSIONS

To our knowledge, this is the first study showing information about disrupted healthcare during the COVID-19 pandemic directly reported by households across several LAC countries, with information over the years 2020 and 2021, and exploring associations with the pandemic situation, policies implemented, and key contextual country factors.

The disruption of healthcare services during the pandemic will likely influence health outcomes in several ways, including decreases in diagnosis (e.g. due to mobility restrictions and constrained health system capacity), increased waiting time for treatment after diagnosis (e.g. due to backlog of diagnosed cases), and potential declines in quality of care (e.g. due to decreased health system capacity). The findings of this study provide relevant information about the magnitude and factors associated with healthcare disruption during the COVID-19 pandemic that can inform decision-making about the recovery of services in the short term and the main considerations for strengthening health system resilience to confront future public health emergencies such as epidemics and pandemics in the longer-term.

## Additional material


Online Supplementary Document
