# Supplementary material for: Healthcare service disruption in 14 Latin American and Caribbean countries during the COVID-19 pandemic: Analysis of household phone surveys, 2020-2021
Source: J Glob Health. 2023 Jul 21;13:06023. doi: 10.7189/jogh.13.06023 (PMC10359759; doi:10.7189/jogh.13.06023)
Supplement: Online Supplementary Document [file jogh-13-06023-s001.pdf]

Supplementary material

Table of Contents

eTable 1. High Frequency Phone Surveys details by country and survey round 2

eTable 2. Reasons for intermittently disrupted healthcare, 4-survey waves trend 2020-2021, 14 LAC countries....4

eFigure 1. COVID-19 new cases and disrupted healthcare in 14 LAC countries, 2020 and 2021. 7

eFigure 2. COVID-19 new cases and disrupted healthcare due to COVID-19 concerns in 14 LAC countries, 2020 and 2021. 8

eFigure 3. COVID-19 Stringency Index and disrupted healthcare in 14 LAC countries, 2020 and 2021. 9

eFigure 4. COVID-19 Stringency Index and disrupted healthcare due to public health measures in 14 LAC countries, 2020 and 2021. 10

eFigure 5. Out-of-pocket health expenditure and disrupted healthcare in 14 LAC countries (average of 4-rounds per country) 11

eFigure 6. Out-of-pocket health spending as percentage of total health spending and disrupted healthcare due to financial concerns in 14 LAC countries, 2020 and 2021 (average of 4-rounds per country). 12

eFigure 7. Hospital beds per 1000 population and disrupted healthcare in 14 LAC countries (average of 4-rounds per country) 13

eFigure 8. Physicians per 1000 population and disrupted healthcare in 14 LAC countries (average of 4-rounds per country) 14

eFigure 9. Nurses per 1000 population and disrupted healthcare in 14 LAC countries (average of 4-rounds per country) 15

eFigure 10. Hospital beds per 1000 population and disrupted healthcare due to healthcare supply constraints in 14 LAC countries, 2020 and 2021 (average of 4-rounds per country). 16

eFigure 11. Physicians per 1000 population and disrupted healthcare due to healthcare supply constraints in 14 LAC countries, 2020 and 2021 (average of 4-rounds per country). 17

eFigure 12. Nurses per 1000 population and disrupted healthcare due to financial concerns in 14 LAC countries, 2020 and 2021 (average of 4-rounds per country). 18

eTable 3. Variance inflation factor analysis correlation matrix for multicollinearity. 18

eTable 4. Ordinary least squares and Logit regression analysis of healthcare disruption in 14 LAC countries. 19

eTable 1. High Frequency Phone Surveys details by country and survey round

Country	Round	Month	% of female respondents	% of rural households	Average age of survey respondent	Average number of household members	Number of households surveyed
Argentina	Round 1	June	60.64%	10.79%	48	4	1001
Argentina	Round 2	July	61.24%	10.66%	49	4	694
Argentina	Round 3	August	59.94%	11.13%	48	3	629
Argentina	Round 4	June	56.99%	10.86%	47	3	1216
Bolivia	Round 1	May	49.77%	25.02%	37	5	1075
Bolivia	Round 2	June	48.51%	25.67%	37	5	670
Bolivia	Round 3	July	47.82%	24.75%	37	5	711
Bolivia	Round 4	May	46.15%	19.03%	36	5	1272
Chile	Round 1	May	54.6%	20.1%	44	4	1000
Chile	Round 2	July	53.38%	20.42%	46	4	622
Chile	Round 3	August	53.8%	20.18%	44	4	684
Chile	Round 4	June	58.5%	16.34%	42	4	1212
Colombia	Round 1	June	60%	25.3%	42	4	1000
Colombia	Round 2	July	61.78%	26.16%	43	4	730
Colombia	Round 3	August	60.03%	25.24%	43	4	638
Colombia	Round 4	June	58.15%	19.25%	41	4	1221
Costa Rica	Round 1	May	51.19%	48.94%	41	4	801
Costa Rica	Round 2	July	51.73%	48.74%	41	4	636
Costa Rica	Round 3	August	52.74%	48.18%	41	4	658
Costa Rica	Round 4	June	57.52%	50.43%	42	4	805
Dominican Republic	Round 1	May	53.16%	17.97%	40	4	807
Dominican Republic	Round 2	July	53.49%	17.24%	40	4	673
Dominican Republic	Round 3	August	53.82%	18.44%	40	4	667

Country	Round	Month	% of female respondents	% of rural households	Average age of survey respondent	Average number of household members	Number of households surveyed
Dominican Republic	Round 4	June	54.19%	33.2%	40	4	1205
Ecuador	Round 1	May	49.47%	23.76%	41	4	1227
Ecuador	Round 2	June	50.44%	22.48%	42	4	1025
Ecuador	Round 3	July	50.06%	23%	42	4	853
Ecuador	Round 4	May	55.55%	25.59%	38	4	1352
El Salvador	Round 1	June	48.38%	-	39	4	804
El Salvador	Round 2	July	48.48%	-	39	4	625
El Salvador	Round 3	August	47.52%	-	39	4	604
El Salvador	Round 4	June	51.59%	35.82%	39	4	818
Guatemala	Round 1	May	49.88%	-	35	5	806
Guatemala	Round 2	July	49.12%	-	36	5	625
Guatemala	Round 3	August	48.43%	-	35	5	636
Guatemala	Round 4	June	50.21%	40.68%	37	5	1207
Honduras	Round 1	June	55.51%	-	36	5	807
Honduras	Round 2	July	53.64%	-	37	5	550
Honduras	Round 3	August	56.43%	-	37	5	521
Honduras	Round 4	July	57%	39.28%	36	5	1021
Mexico	Round 1	June	59.46%	20.29%	45	4	2109
Mexico	Round 2	July	59.28%	18.15%	47	4	1245
Mexico	Round 3	August	58.21%	17.7%	48	4	1175
Mexico	Round 4	June	56.61%	18.86%	45	4	2625
Paraguay	Round 1	June	53.01%	21.54%	37	5	715
Paraguay	Round 2	July	51.44%	21.4%	37	5	486
Paraguay	Round 3	August	52.52%	21.66%	37	5	457
Paraguay	Round 4	June	53.81%	18.4%	38	4	1076

**Country**	**Round**	**Month**	**% of female respondents**	**% of rural households**	**Average age of survey respondent**	**Average number of household members**	**Number of households surveyed**
Peru	Round 1	May	52.1%	23.4%	38	5	1000
Peru	Round 2	June	52.44%	22.35%	38	5	841
Peru	Round 3	July	51.89%	21.56%	38	5	821
Peru	Round 4	June	54.79%	25.08%	38	5	1212
St. Lucia	Round 1	May	54.53%	43.46%	54	3	1093
St. Lucia	Round 2	August	53.23%	43.22%	50	3	900
St. Lucia	Round 4	June	55.33%	50.9%	41	4	835

eTable 2. Reasons for intermittently disrupted healthcare, 4-survey waves trend 2020-2021, 14 LAC countries

**Round / Reasons**	**All reasons**	**Concerns about contracting COVID-19**	**Financial concerns**	**Healthcare supply constraints**	**Public health measures**	**Other reason**
Ecuador						
Round 1	44.92%	13.98%	0.64%	17.16%	5.93%	7.2%
Round 2	25.3%	7.32%	2.44%	9.76%	2.13%	3.66%
Round 3	20.89%	4.11%	2.22%	9.49%	1.58%	3.48%
Round 4	11.52%	0.77%	0.77%	8.25%	0.58%	1.34%
Peru						
Round 1	40.96%	9.94%	0.6%	23.19%	5.12%	2.11%
Round 2	22.48%	5.96%	3.21%	12.39%	0.92%	0%
Round 3	22.62%	5.95%	2.38%	13.1%	0.4%	0.79%
Round 4	4.92%	0.2%	0.61%	3.28%	0%	1.02%
Bolivia						
Round 1	36.13%	9.35%	1.29%	11.94%	8.06%	5.48%
Round 2	30.91%	9.7%	1.82%	13.94%	3.64%	1.82%
Round 3	25.81%	6.45%	0.46%	15.21%	1.84%	1.84%

Round / Reasons	All reasons	Concerns about contracting COVID-19	Financial concerns	Healthcare supply constraints	Public health measures	Other reason
Round 4	3.5%	0.7%	0%	1.86%	0.23%	0.7%
Paraguay						
Round 1	27.14%	6.67%	0.95%	11.43%	7.14%	0.95%
Round 2	13.68%	1.71%	2.56%	6.84%	1.71%	0.85%
Round 3	12.5%	2.5%	1.88%	5%	1.88%	1.25%
Round 4	4.46%	1.05%	1.05%	1.57%	0.52%	0.26%
Dominican Republic						
Round 1	26.42%	7.32%	1.22%	12.6%	3.25%	2.03%
Round 2	11.72%	3.77%	2.51%	5.02%	0%	0.42%
Round 3	15.91%	7.2%	3.41%	5.3%	0%	0%
Round 4	1.41%	0%	0.85%	0.28%	0%	0.28%
El Salvador						
Round 1	24.91%	5.8%	0.34%	9.9%	5.8%	3.07%
Round 2	14.81%	3.7%	2.12%	7.41%	1.06%	0.53%
Round 3	8.96%	1.49%	1%	2.99%	2.99%	0.5%
Round 4	0.39%	0%	0%	0%	0.39%	0%
Colombia						
Round 1	24.32%	2.98%	0.25%	15.38%	4.96%	0.74%
Round 2	8.61%	0.48%	0.48%	6.22%	1.44%	0%
Round 3	7.59%	0.42%	0%	6.75%	0.42%	0%
Round 4	6.99%	0.42%	1.27%	3.18%	0.64%	1.48%
St. Lucia						
Round 1	23.3%	2.27%	6.82%	10.8%	2.84%	0.57%
Round 2	6.21%	0%	3.92%	0.65%	0.33%	1.31%
Round 4	2.58%	0%	0%	0.52%	0%	2.06%

Round / Reasons	All reasons	Concerns about contracting COVID-19	Financial concerns	Healthcare supply constraints	Public health measures	Other reason
Chile						
Round 1	20.53%	4.27%	0.27%	10.13%	0.8%	5.07%
Round 2	7.96%	1.99%	0%	2.99%	1%	1.99%
Round 3	6.94%	1.85%	0%	2.78%	0.46%	1.85%
Round 4	1.5%	0%	0%	1.31%	0%	0.19%
Honduras						
Round 1	17.97%	1.38%	2.3%	5.99%	5.99%	2.3%
Round 2	3.79%	0.76%	0.76%	0.76%	1.52%	0%
Round 3	7.59%	1.38%	1.38%	4.14%	0.69%	0%
Round 4	0.31%	0%	0%	0%	0%	0.31%
Guatemala						
Round 1	17.42%	1.69%	3.93%	6.18%	5.06%	0.56%
Round 2	8.61%	1.32%	2.65%	3.31%	1.32%	0%
Round 3	9.41%	1.18%	6.47%	0.59%	1.18%	0%
Round 4	1.16%	0%	0.78%	0%	0.39%	0%
Argentina						
Round 1	11.89%	1.83%	0.3%	6.1%	3.05%	0.61%
Round 2	4.15%	1.04%	0%	3.11%	0%	0%
Round 3	5.81%	0.58%	0%	3.49%	1.16%	0.58%
Round 4	2.03%	0%	0%	1.69%	0%	0.34%
Mexico						
Round 1	11.62%	2.04%	0.63%	6.59%	0.78%	1.57%
Round 2	2.64%	0.33%	0%	1.65%	0.33%	0.33%
Round 3	2.87%	0.57%	0.57%	1.43%	0%	0.29%
Round 4	0.6%	0%	0%	0.6%	0%	0%

Round / Reasons	All reasons	Concerns about contracting COVID-19	Financial concerns	Healthcare supply constraints	Public health measures	Other reason
Costa Rica						
Round 1	9.74%	1.3%	0%	3.9%	0.97%	3.57%
Round 2	4.31%	0.96%	0%	2.87%	0%	0.48%
Round 3	5.24%	0.48%	2.38%	1.9%	0%	0.48%
Round 4	0.36%	0%	0%	0.36%	0%	0%

eFigure 1. COVID-19 new cases and disrupted healthcare in 14 LAC countries, 2020 and 2021.

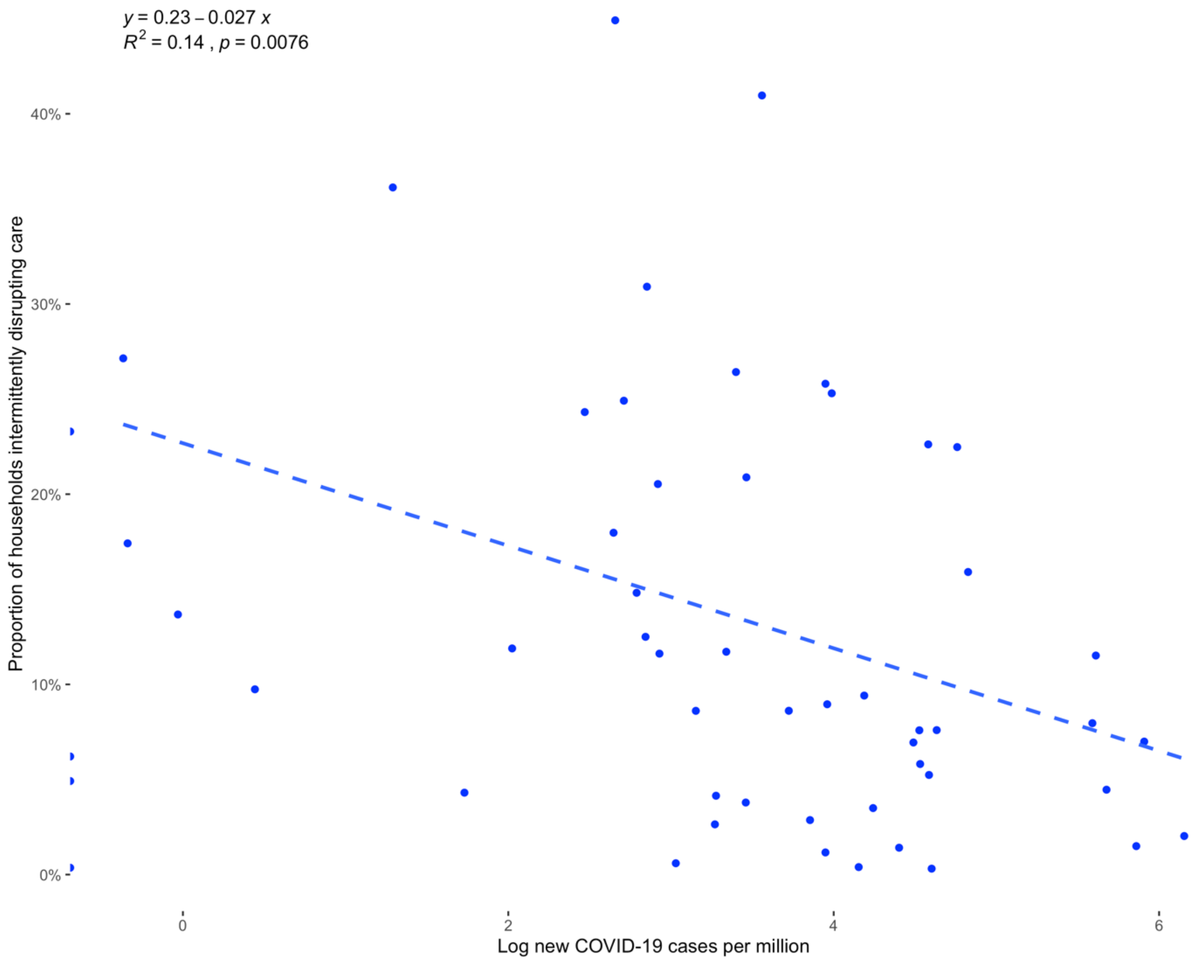

eFigure 2. COVID-19 new cases and disrupted healthcare due to COVID-19 concerns in 14 LAC countries, 2020 and 2021.

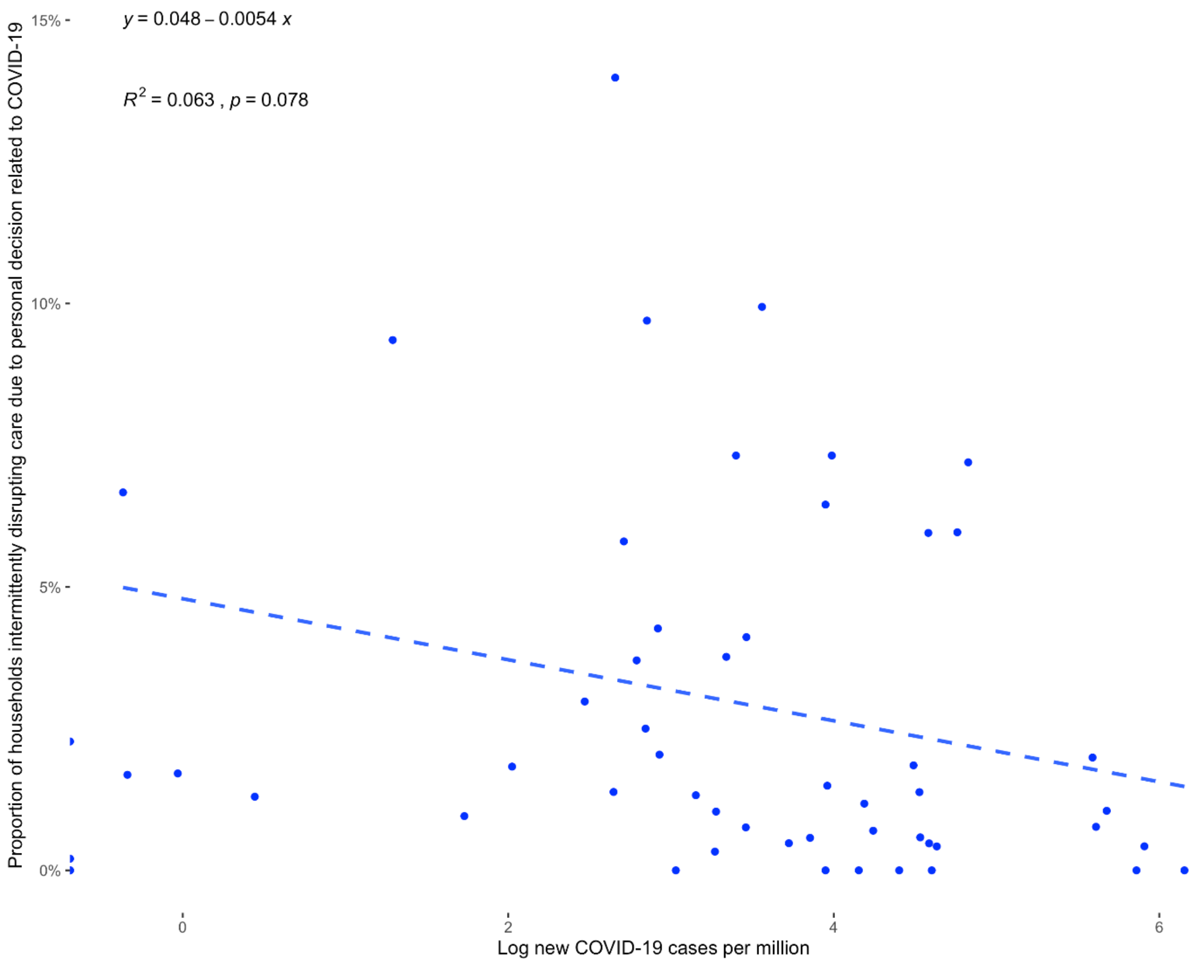

eFigure 3. COVID-19 Stringency Index and disrupted healthcare in 14 LAC countries, 2020 and 2021.

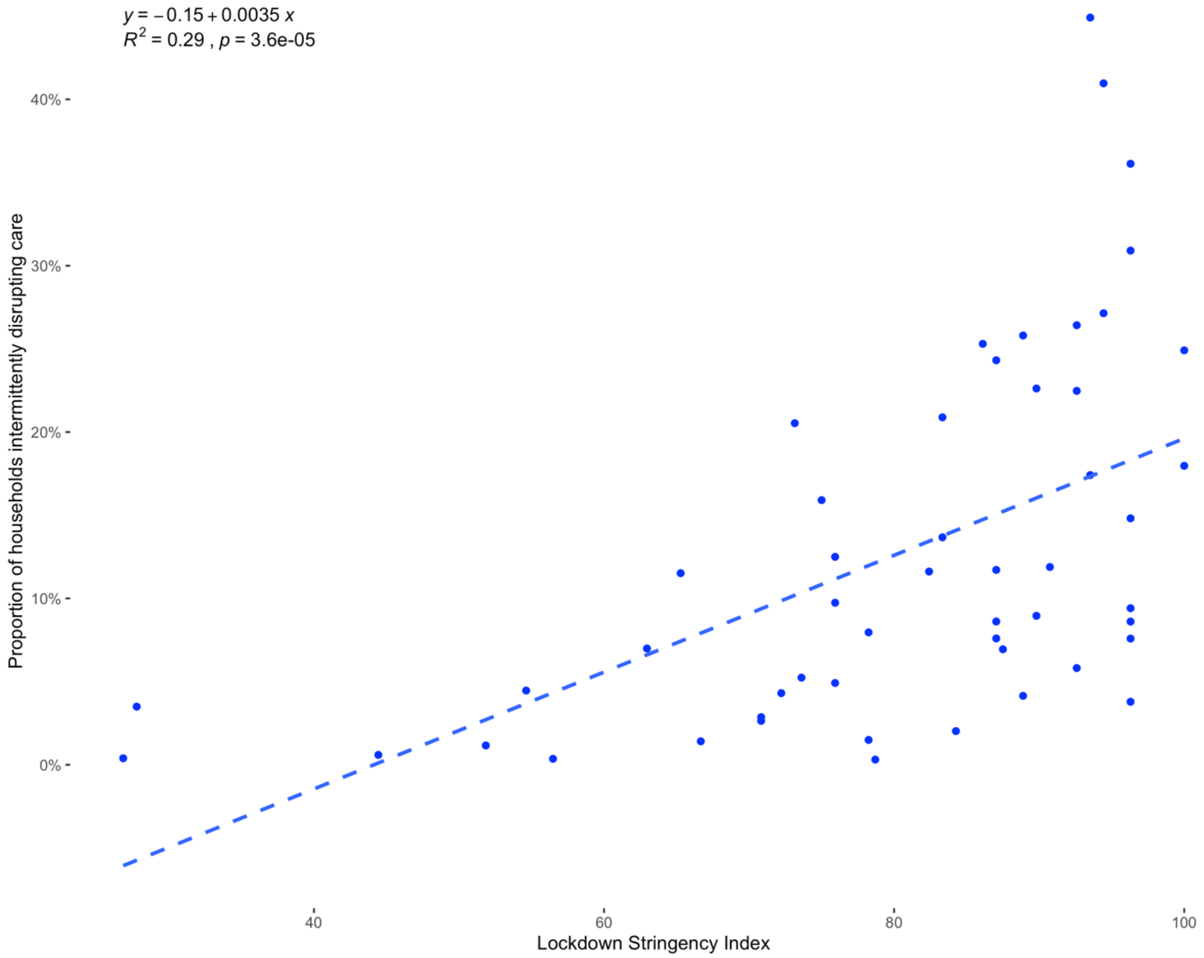

eFigure 4. COVID-19 Stringency Index and disrupted healthcare due to public health measures in 14 LAC countries, 2020 and 2021.

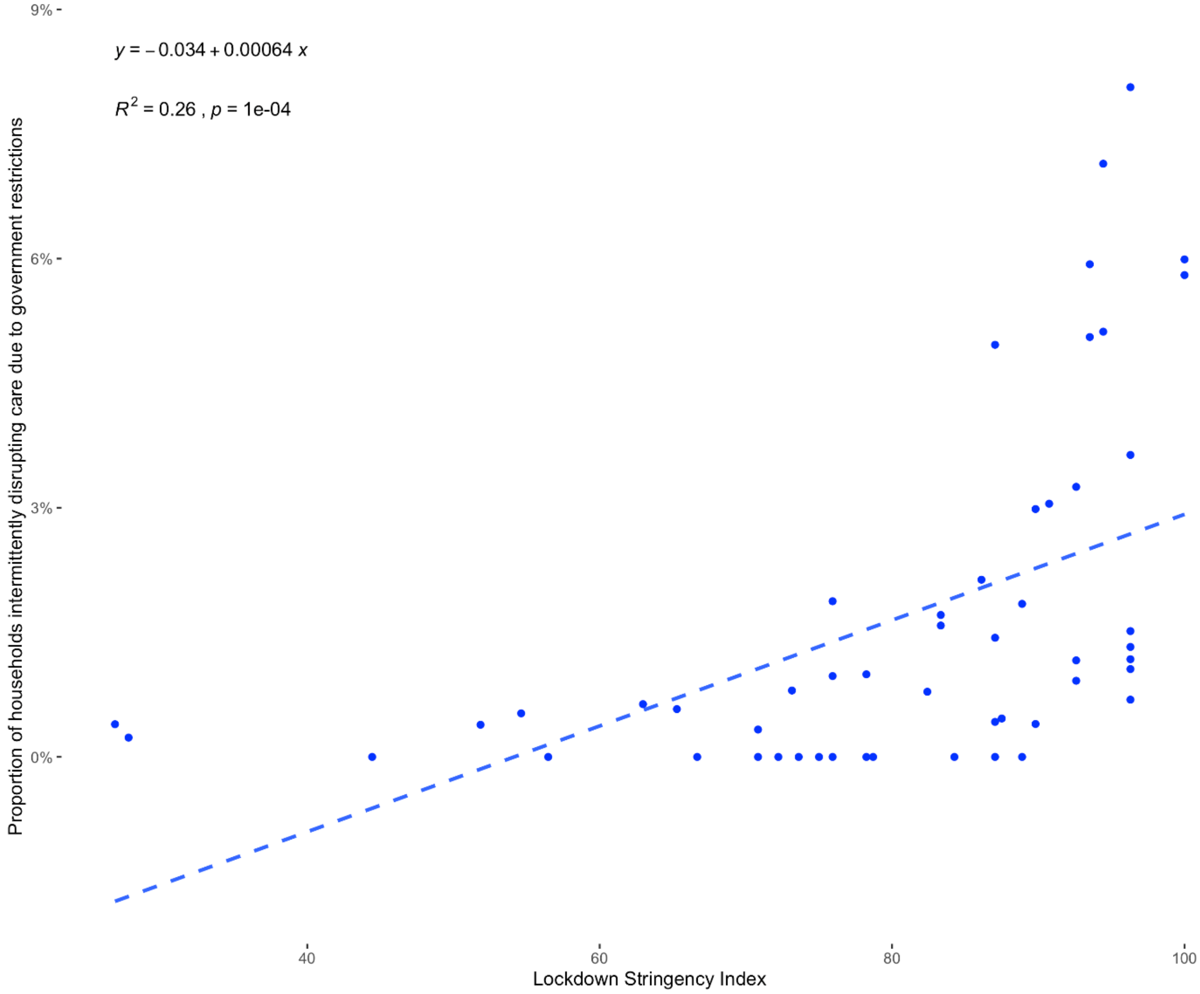

eFigure 5. Out-of-pocket health expenditure and disrupted healthcare in 14 LAC countries (average of 4-rounds per country)

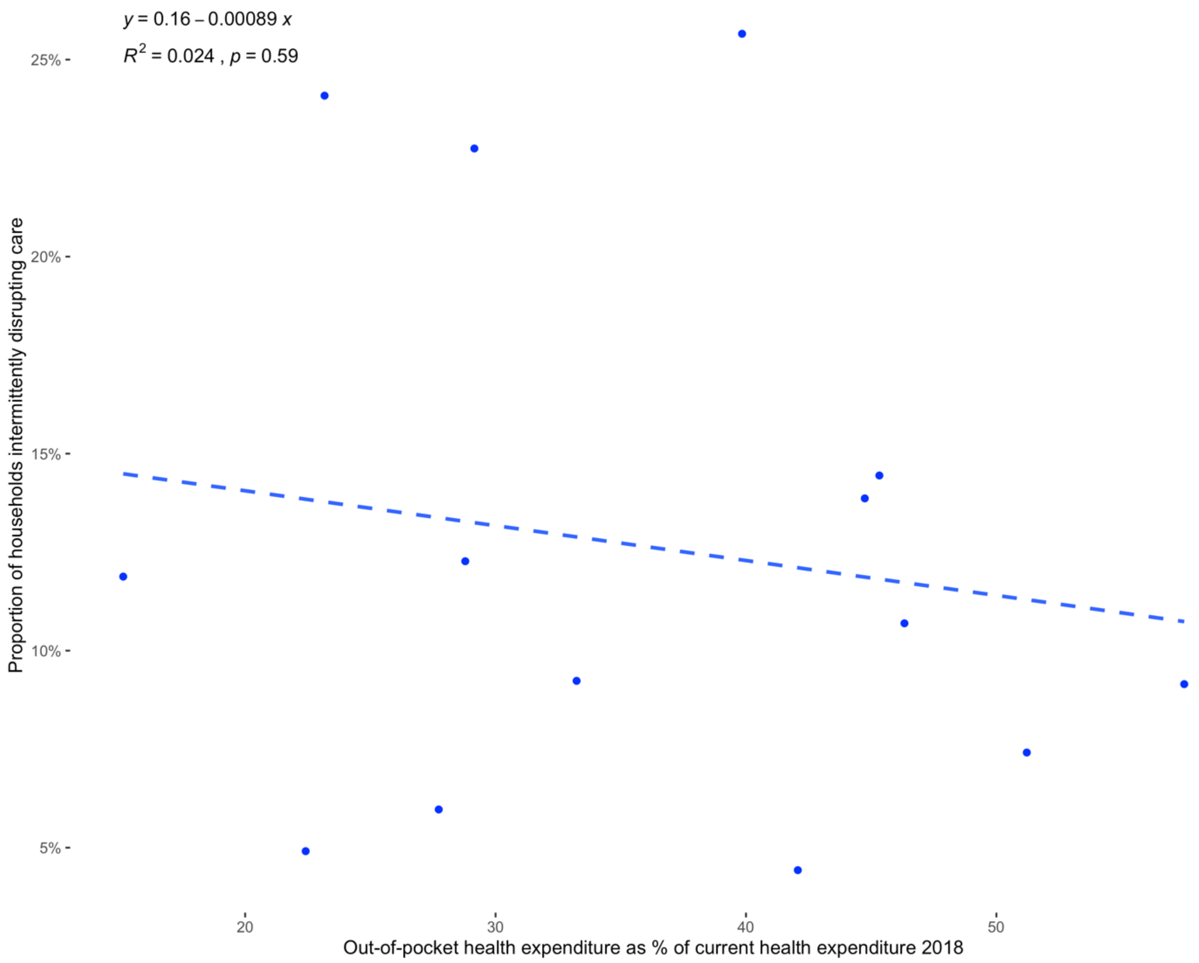

eFigure 6. Out-of-pocket health spending as percentage of total health spending and disrupted healthcare due to financial concerns in 14 LAC countries, 2020 and 2021 (average of 4-rounds per country).

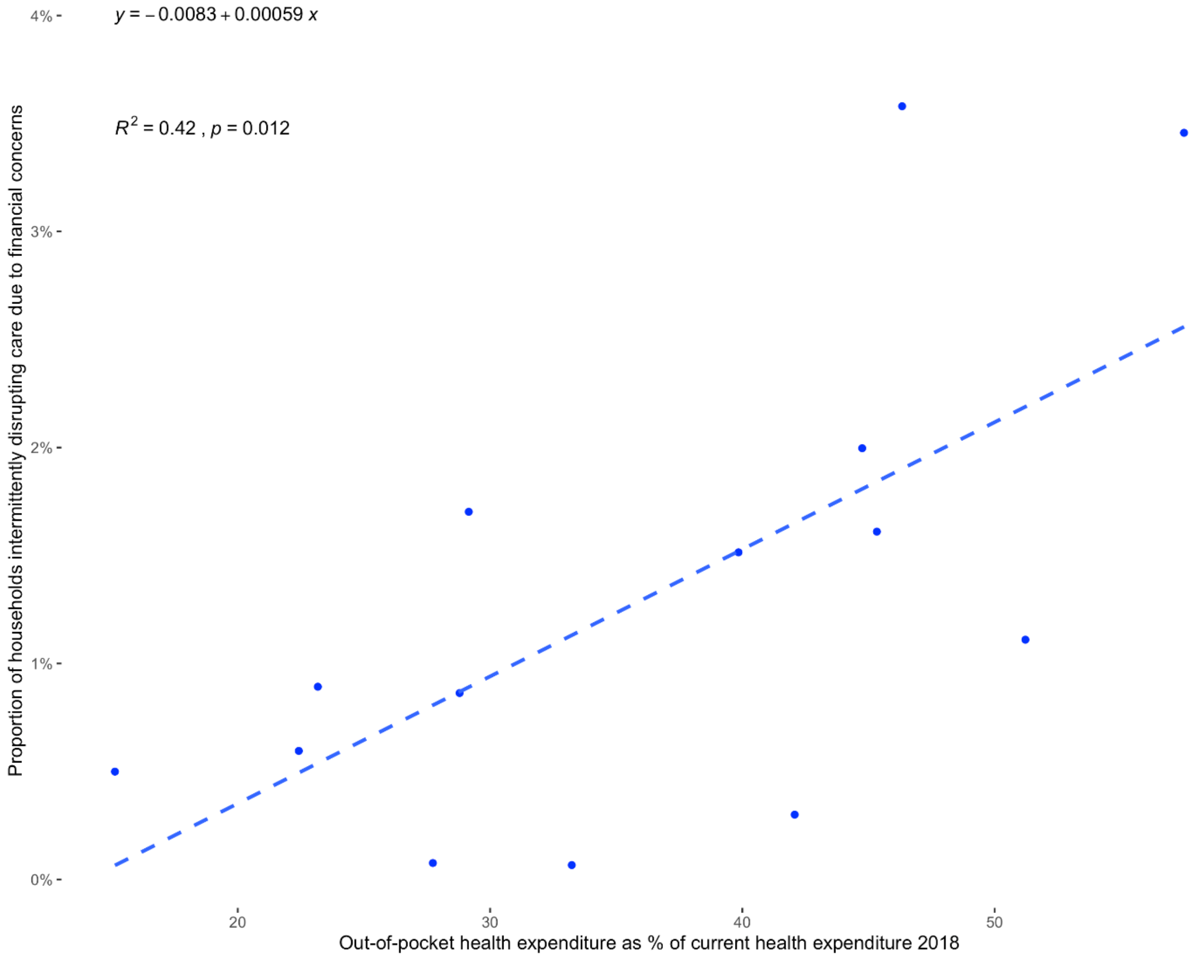

eFigure 7. Hospital beds per 1000 population and disrupted healthcare in 14 LAC countries (average of 4-rounds per country)

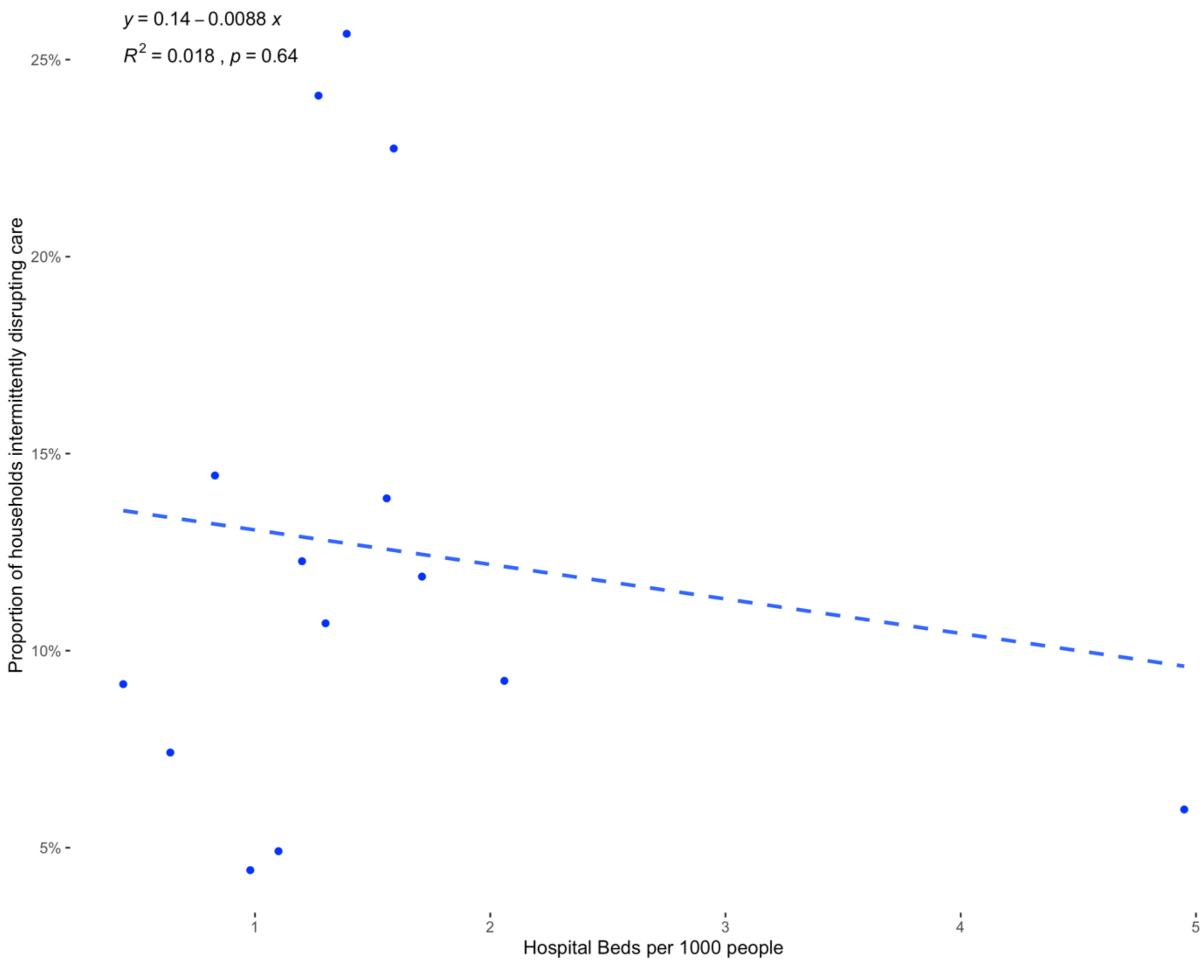

eFigure 8. Physicians per 1000 population and disrupted healthcare in 14 LAC countries (average of 4-rounds per country)

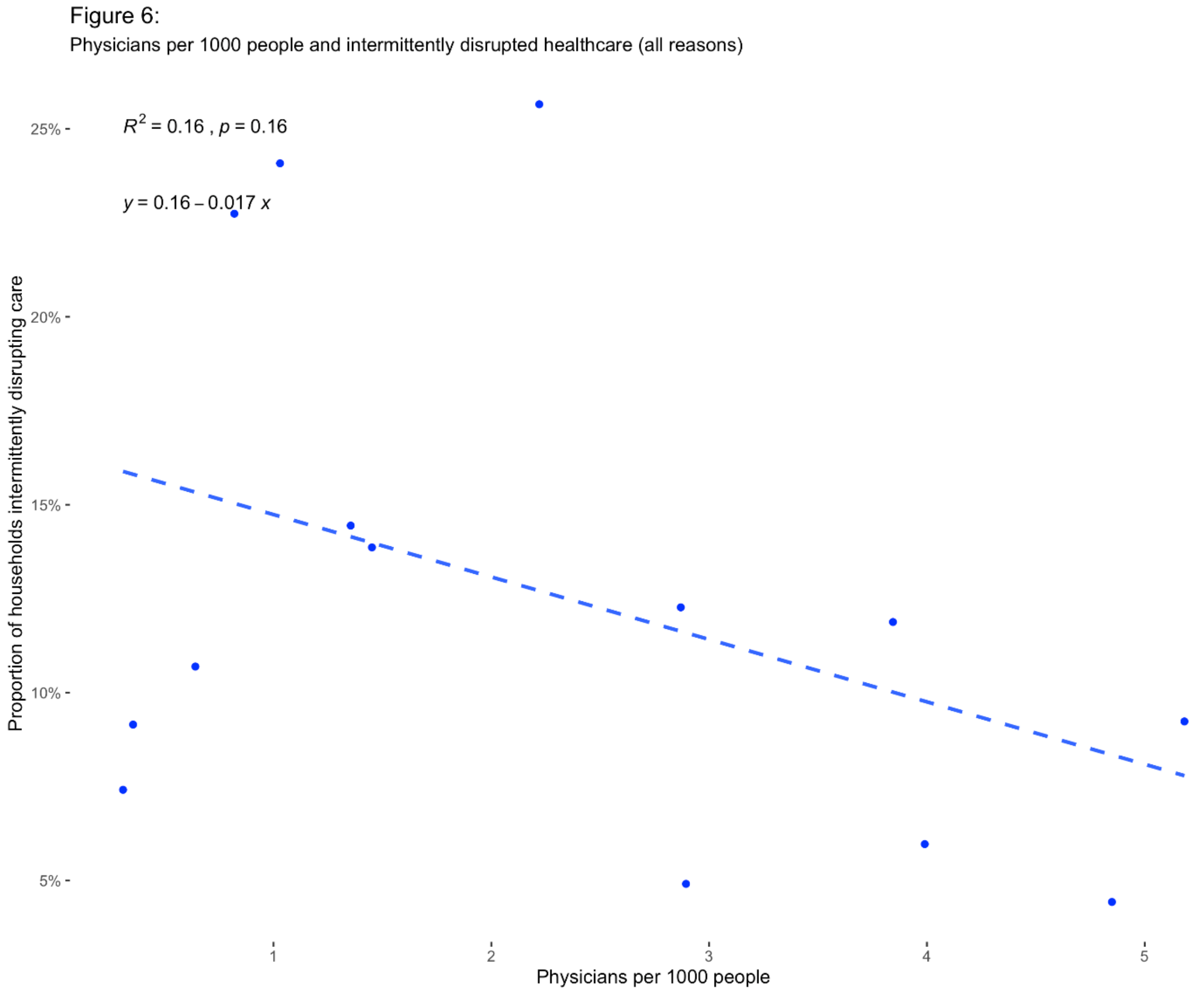

eFigure 9. Nurses per 1000 population and disrupted healthcare in 14 LAC countries (average of 4-rounds per country)

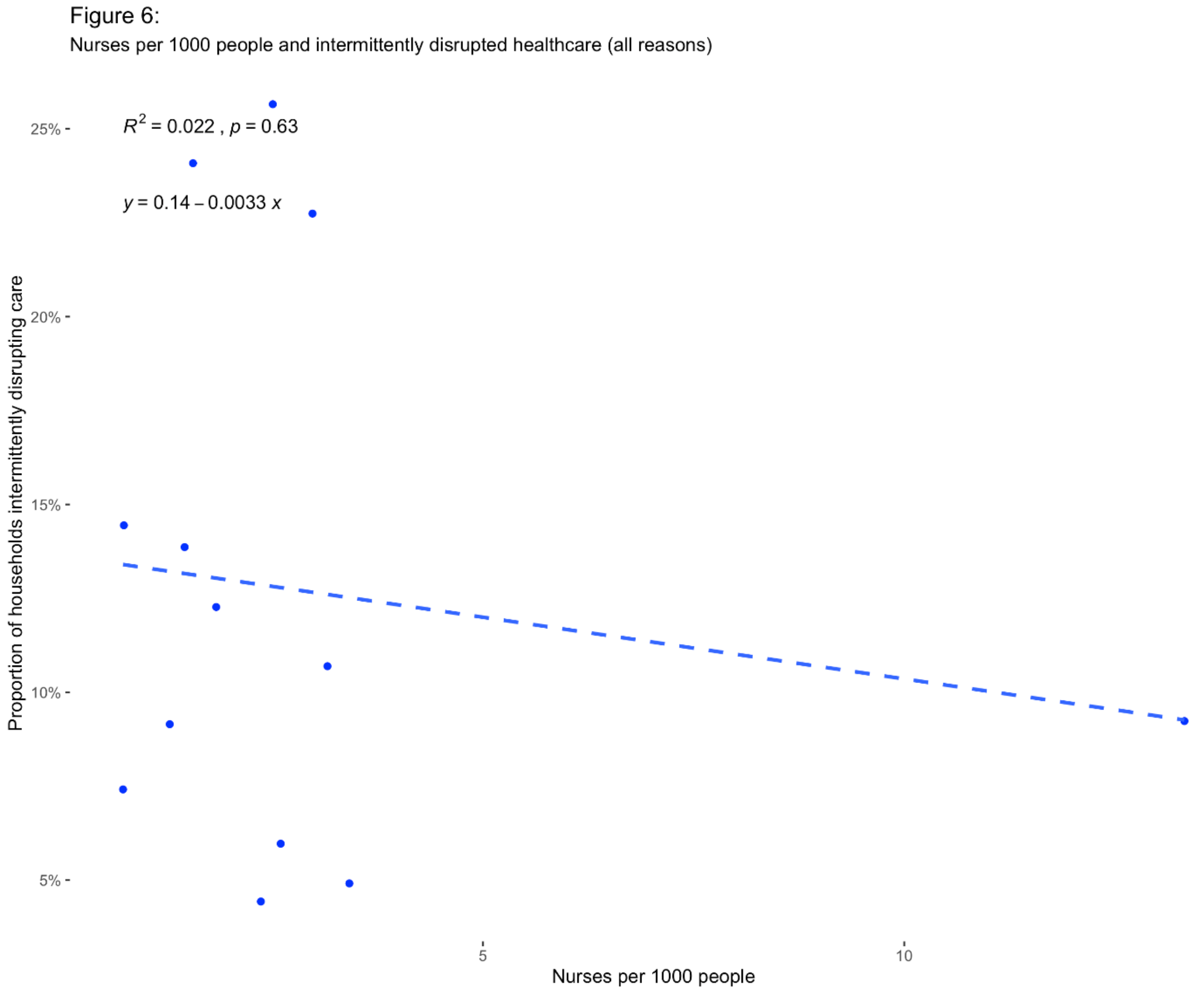

eFigure 10. Hospital beds per 1000 population and disrupted healthcare due to healthcare supply constraints in 14 LAC countries, 2020 and 2021 (average of 4-rounds per country).

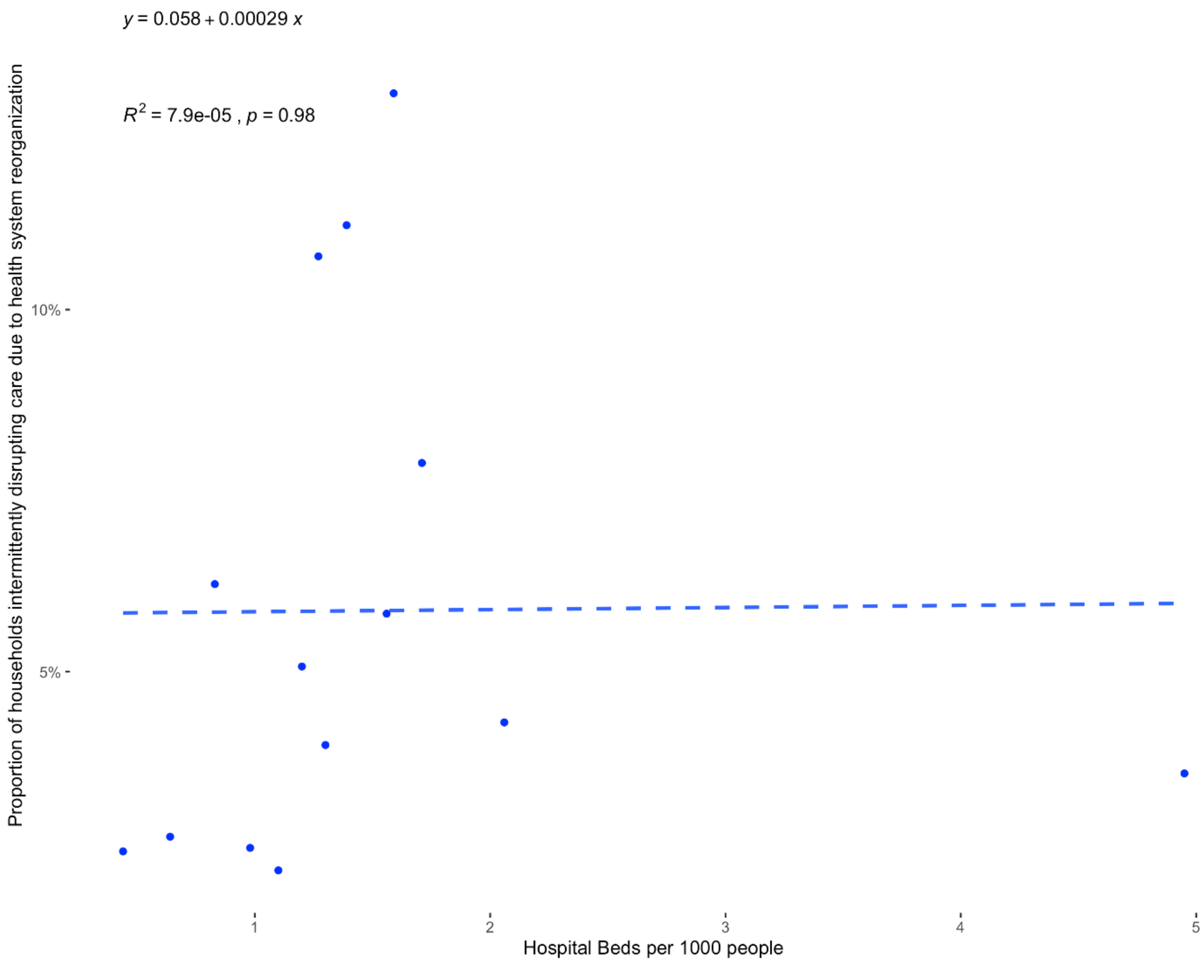

eFigure 11. Physicians per 1000 population and disrupted healthcare due to healthcare supply constraints in 14 LAC countries, 2020 and 2021 (average of 4-rounds per country).

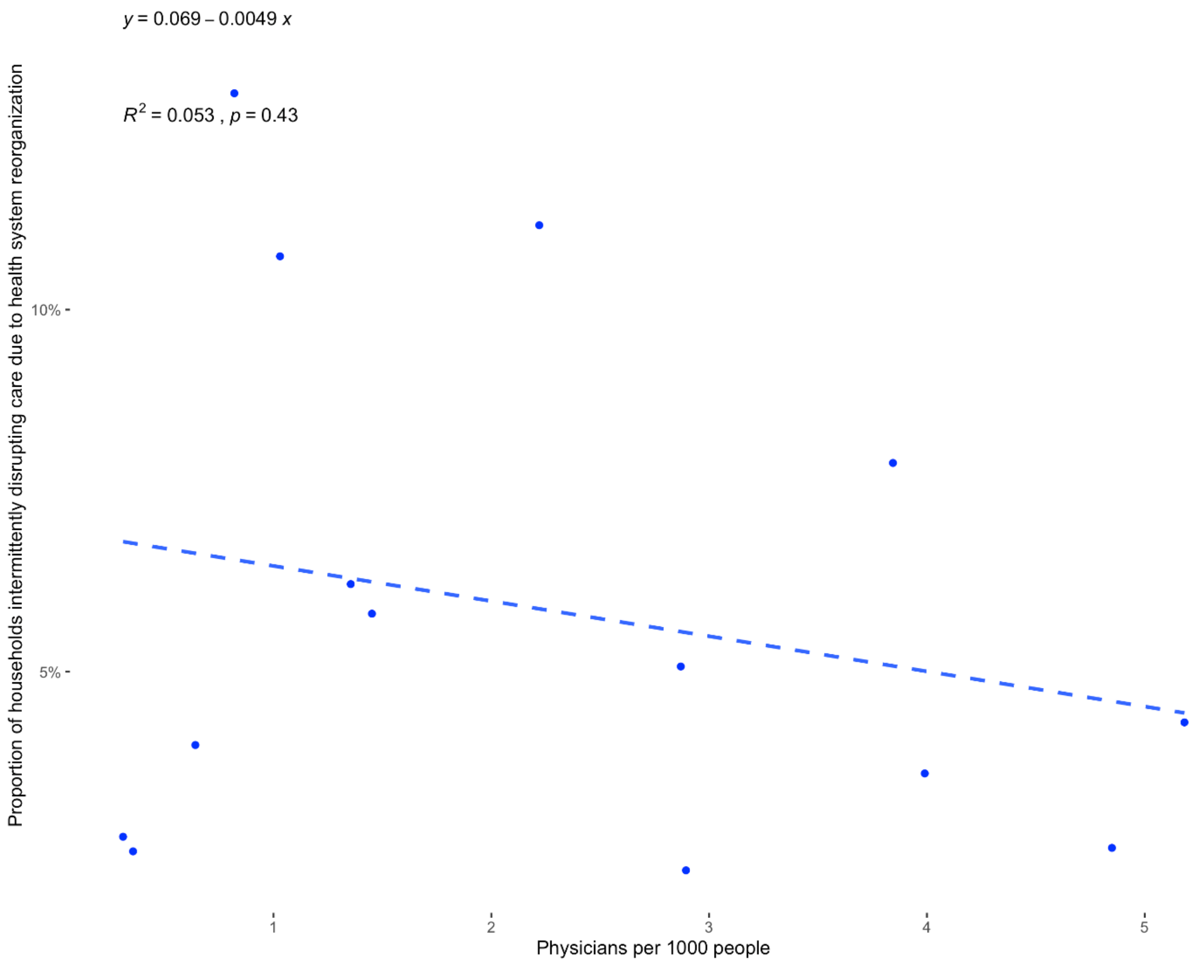

eFigure 12. Nurses per 1000 population and disrupted healthcare due to financial concerns in 14 LAC countries, 2020 and 2021 (average of 4-rounds per country).

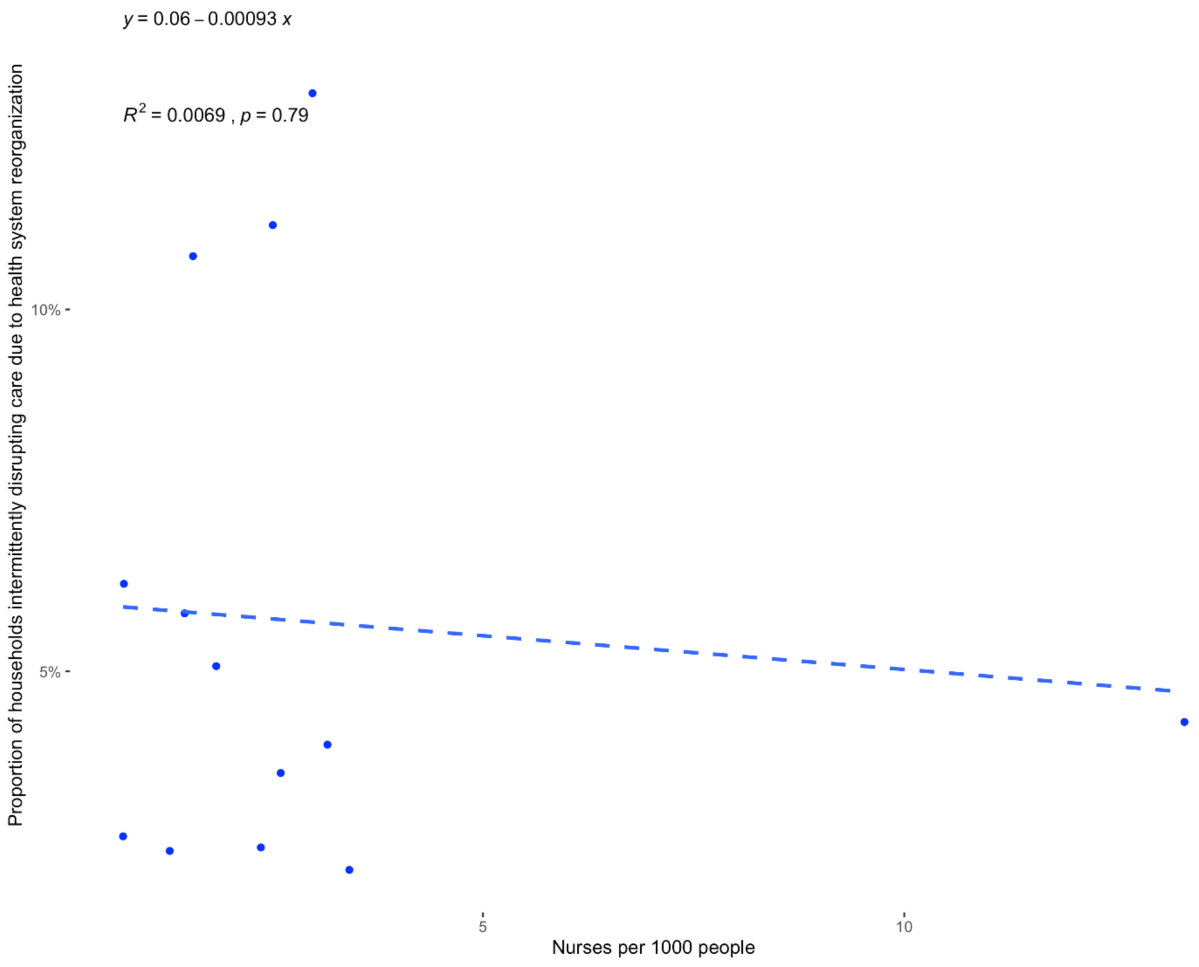

eTable 3. Variance inflation factor analysis correlation matrix for multicollinearity.

	Log COVID-19 new cases per million	Oxford COVID-19 stringency index	GDP per capita (current US$) 2020	Out-of-pocket health expenditure as % of current health expenditure 2018	Hospital beds per 1000 population	Physicians per 1000 population	Nurses per 1000 population	Population density (people per sq. km of land area) 2020	Rural population (% of total population) 2020	Age dependency ratio (% of working-age population) 2020
Log COVID-19 new cases per million	1									
Oxford COVID-19 stringency index	-0.2658	1								

GDP per capita (current US$) 2020	0.0977	-0.0553	1							
Out-of-pocket health expenditure as % of current health expenditure 2018	0.0036	0.0158	-0.2598	1						
Hospital beds per 1000 population	0.1924	0.182	0.3525	-0.449	1					
Physicians per 1000 population	0.087	-0.1676	0.7573	-0.2536	0.366	1				
Nurses per 1000 population	0.2634	0.0143	0.7634	-0.2509	0.2418	0.5997	1			
Population density (people per sq. km of land area) 2020	-0.0589	0.0086	-0.2406	0.1939	-0.268	-0.1725	-0.2755	1		
Rural population (% of total population) 2020	-0.1483	0.0266	-0.7383	0.5454	-0.6535	-0.719	-0.4953	0.1205	1	
Age dependency ratio (% of working-age population) 2020	-0.1058	0.0312	-0.8119	0.2604	-0.0596	-0.6552	-0.6321	0.1253	0.6069	1

eTable 4. Ordinary least squares and Logit regression analysis of healthcare disruption in 14 LAC countries.

**Variables**	**OLS model all variables**	**OLS model after VIF**
	Health care not received	Health care not received
Log new COVID-19 cases per million population	0.01545***	0.01846***
	(0.00365)	(0.00318)
Oxford COVID-19 stringency index	0.00147***	0.00155***
	(0.00027)	(0.00023)
Hospital beds per 1000 population	-0.00255	-0.01126***
	(0.00574)	(0.00276)
Out-of-pocket health expenditure as % of current health expenditure 2018	-0.00140***	-0.00077***
	(0.00046)	(0.00028)
GDP per capita (current US$) 2020	-0.00002***	-0.00001***
	(0.00000)	(0.00000)
Population density (people per sq. km of land area) 2020	-0.00014***	-0.00012***
	(0.00004)	(0.00004)
Physicians per 1000 population	-0.00266	
	(0.00336)	

Nurses per 1000 population	0.00297**	
	(0.00140)	
Rural population (% of total population) 2020	0.00065	
	(0.00070)	
Age dependency ratio (% of working-age population) 2020	-0.00460***	
	(0.00165)	
_cons	0.39594***	0.08045***
	(0.09682)	(0.01726)
N	13044	14365
R-sq	0.105	0.097

**Variables**	**Logit model all variables**	**Logit model after VIF**
	Health care not received	Health care not received
Log new COVID-19 cases per million population	0.18231***	0.01711***
	(0.03602)	(0.00369)
Oxford COVID-19 stringency index	0.01317***	-0.00010***
	(0.00405)	(0.00001)
Hospital beds per 1000	0.06817	-0.07813**
	(0.05105)	(0.03495)
Out-of-pocket health expenditure as % of current health expenditure 2018	-0.01442***	-0.00762***
	(0.00398)	(0.00258)
GDP per capita (current US$) 2020	-0.00009***	-0.00010***
	(0.00003)	(0.00001)
Population density (people per sq. km of land area) 2020	-0.00044	-0.00100***
	(0.00039)	(0.00036)
Physicians per 1000 population	-0.03567	
	(0.03283)	
Nurses per 1000 population	0.02509	
	(0.01608)	
Rural population (% of total population) 2020	0.02258***	
	(0.00663)	
Age dependency ratio (% of working-age population) 2020	-0.01999	
	(0.01225)	
1.year_month_num	0.00000	0.00000
	(.)	(.)
2.year_month_num	-0.74998***	-0.79036***
	(0.08647)	(0.07334)
3.year_month_num	-1.29249***	-1.38193***

	(0.09664)	(0.09044)
4.year_month_num	-1.71868***	-1.85739***
	(0.13041)	(0.11553)
5.year_month_num	-1.93888***	-1.77428***
	(0.26594)	(0.24961)
6.year_month_num	-3.14693***	-2.86394***
	(0.22691)	(0.19212)
7.year_month_num	-5.64421***	-5.57473***
	(1.01205)	(1.01125)
_cons	-0.89092	-1.65937***
	(0.84674)	(0.34898)
N	13044	14365
R-sq		
